# Emergency visits among end-of-life cancer patients in Taiwan: a nationwide population-based study

**DOI:** 10.1186/s12904-015-0016-0

**Published:** 2015-05-09

**Authors:** Yi-Hui Lee, Dachen Chu, Nan-Ping Yang, Chien-Lung Chan, Shun-Ping Cheng, Jih-Tung Pai, Nien-Tzu Chang

**Affiliations:** School of Nursing, College of Medicine, National Taiwan University, Taipei, Taiwan; Department of Nursing, School of Nursing, College of Medicine, Chang-Gang University, Taoyuan, Taiwan; Institute of Public Health, College of Medicine, National Yang-Ming University, Taipei, Taiwan; Department of Neurosurgery, Taipei City Hospital, Taipei, Taiwan; Department of Medical Research, Keelung General Hospital, Ministry of Health & Welfare, Keelung, Taiwan; Department of Information Management, Yuan-Ze University, Taoyuan, Taiwan; Department of Rehabilitation, Taoyuan General Hospital, Ministry of Health & Welfare, Taoyuan, Taiwan; Department of Oncology & Internal Medicine, Taoyuan General Hospital, Ministry of Health & Welfare, Taoyuan, Taiwan

**Keywords:** Cancer, Emergency visits, End-of-Life

## Abstract

**Background:**

An increased number of emergency visits at the end of life may indicate poor-quality cancer care. The study aimed to investigate the prevalence and utilization of emergency visits and to explore the reasons for emergency department (ED) visits among cancer patients at the end of life.

**Methods:**

A retrospective cohort study was performed by tracking one year of ambulatory medical service records before death. Data were collected from the cancer dataset of Taiwan’s National Health Insurance Research Database (NHIRD).

**Results:**

A total of 32,772 (19.2%) patients with malignant cancer visited EDs, and 23,883 patients died during the study period. Of these, the prevalence of emergency visits in the mortality group was 81.5%, and their ED utilization was significantly increased monthly to the end of life. The most frequent types of cancer were digestive and peritoneum cancers (34.8%), followed by breast cancer (17.7%) and head and neck cancers (13.3%). Older patients, males, and those diagnosed with metastases, respiratory or digestive cancer were more likely to use ED services at the end of life. Use of an ED service in the nearest community hospital to replace medical centers for dying cancer patients would be more acceptable in emergency situations.

**Conclusions:**

Our study provided population-based evidence related to ED utilization. An understanding of the reasons for such visits could be useful in preventing overuse of ED visits to improve the quality of end-of-life care.

## Background

Cancer is the first leading cause of death in Taiwan and worldwide. 8.2 million people died from cancer in 2012, and the number of new annual cases will continue to increase by about 70% over the next 2 decades [1]. In the aging Taiwan society, there is also an increasing number of individuals dying of cancer who are covered by the national health insurance (NHI) system. Approximately 29,000 Taiwanese people died from cancer in 1997, and this increased to 40,300 in 2007, which accounted for 13 percent of deaths attributable to cancer [2,3]. There is a growing appreciation of cancer patients’ needs for end-of-life (EOL) care services, especially for those with terminal illnesses. Cancer care tends to be aggressive in Taiwan, as shown by the increasing anticancer therapies, ICU stays, and hospital deaths [4,5]. Patients were found to be more likely to receive aggressive care if they were younger or lived in rural regions with fewer medical resources [6-8]. However, this might go against old Taiwanese beliefs. In Taiwan, “Dying in his/her own home is a good death” when it comes to the most important issue for terminal patients and their families in Taiwanese culture [9]. To respect the end of life medical wishes of patients, the Taiwan Hospice-Palliative Care Act was initiated in 2000. Its purpose was to help incurable patients to relieve their physical, psychological and spiritual suffering by palliative or supportive medical care, and it permits the legal termination of end-of-life patients’ life-supporting devices based on safeguarding the rights and wills of these patients [10]. Whereas the NHI in Taiwan issues reimbursement to cancer patients who have the right to refuse or receive aggressive medical treatment, the economic burden of cancer treatment in intensive care units presents an increasing trend [11].

For dying cancer patients, aggressive medical treatments and emergency department (ED) visits can result in distress and exhaustion. Abdominal pain, lung cancer, and dyspnea were reported to be the most common reasons for ED visits during the final six months of life and the final two weeks of life in Canada [12]. However, the available information is insufficient due to previous studies focusing on the experiences of these patients who visit an ED near the end of life being limited and having small sample sizes. Taiwan national claims data linked to the death registry database have been used in a similar field, but consideration of the aggressive treatment in EDs during the end of life and related factors were the focus of this study, which was to the best of our knowledge the first of its kind. The quality of EOL care received by terminal cancer patients and the affected factors have also seldom been explored in Asian countries [13]. Requesting an intensive ED service at the EOL stage will not improve the quality of EOL or prolong a patient’s life, and also goes against the traditional beliefs regarding a good death in Taiwan. Therefore, the causes, symptoms and unavoidable events that lead to a terminal cancer patient visiting the emergency department deserve more attention. Reduction of futile aggressive medical treatment needs to be emphasized in order to improve the quality of EOL care [14,15]. To our knowledge, little is known about nationwide ED visits by terminal cancer patients when they are near the end of life [16]. Using Taiwan national claims data linked to the death registry database could be informative. In order to respond to current and emerging health issues apidly and effectively, the Taiwan National Health Insurance Bureau (NHIB) established a uniform system to control the quality of medical services and the coding database. The coverage of this single-payer program is greater than 99%. This nationwide research database contains cancer registration and original claims data for reimbursement, enabling analysis by researchers. The study aimed to explore the prevalence and ED utilizations of all cancer patients in Taiwan in order to reveal the associations between cancer terminal patients and increased utilization of emergency visits near the end-of-life and to compare the distributions of diagnostic problems at EDs between cancer survivors and decedents.

## Methods

### Data source, security, and quality control

Taiwan launched a single-payer National Health Insurance (NHI) Program, financed jointly by payroll taxes, subsidies, and individual premiums, in 1995, and its coverage rate expanded to approximately 99% of the 23 million people in Taiwan who were enrolled in the NHI Research Database. All enrollees enjoy almost free access to healthcare, with a small co-payment by most clinics and hospitals [17,18]. The NHI Research Database includes nationwide population-based data with good quality control and representation and is provided to scientists in Taiwan for research purposes [14,19]. The information related to all subjects is encrypted using a double scrambling protocol for research purposes to protect the privacy of patients. Theoretically, it is impossible to query the data alone to identify individuals at any level using this database. All researchers who wish to use the NHIRD are required to sign a written agreement declaring that they have no intention of attempting to obtain information that could potentially violate the privacy of patients or care providers.

The study design was a retrospective cohort study. The protocol was evaluated by the NHRI (Application Number: 101020), who gave their agreement to the planned analysis of the NHIRD. The data protection and permission protocols were also approved by the Institutional Review Board (IRB) of Taoyuan General Hospital, which has been certificated by the Department of Health, Taiwan (IRB Approval Number: TYGH100021).

### Inclusion and exclusion criteria of the study population and definition

In order to focus on ED utilization among end-of-life malignant cancer patients, the following criteria were identified: (1) emergency cases were excluded if they had used the services of emergency departments but their insurance numbers were not valid for tracing. (2) Patients with malignant cancer were diagnosed according to the International Classification of Diseases, Ninth Revision, Clinical Modification (ICD-9-CM), with first three-digit codes ranging from 140 to 208. The diagnosis codes of cancer types were grouped into six major categories. Their ICD-9-CM codes were defined as described in Table 1. (3) To find the death dates of subjects, another registered catastrophic illness dataset was linked to identify the survival status of the target population and perform the analyses [17]. (4) ED utilization of those with a diagnosis of cancer was identified from the NHIRD, confirmed by the case type code [20]. The definition of end-of-life is: “Any patient who has contracted a serious illness, diagnosed by a physician as at an incurable stage, and dying within 6 months, whose illness will inevitably lead to death in the near future”. The study variable “urban and rural” was defined according to the degree of rurality, which was calculated by the Taiwan NIH research institute by employing parameters used in rurality analysis, which include population density (less than the mean per km^2^), the proportion%) of elderly residents in the whole population, and proportion of actual workers in the area population [21]. The severity of each ED visiting was presented by triage scale.

**Table 1:** **Characteristics of cancer patients in Taiwan, 2008 (n = 170,271)**

	**All pat. (** ***n*** **= 170,271)**	**ED visitors (** ***n*** **= 32,772)**		**Mortality of ED visitors (n =23,883)**	
Age, yr,					
Mean (S.D.)	61.0 (14.9)	64.0	(21.5)	67.0	(14.4)
Gender, No. (%)					
Male	84215 (49.5)	20037	23.8	15551	18.5
Female	86056 (50.5)	12735	14.8	8332	9.7
Types of cancer (ICD_9 codes)					
Head and neck (140-149,160,161)	22600	4088	18.1	2819	12.5
Digestive and peritoneum (150-159)	59223	15568	26.3	12245	20.7
Colorectal (153-154)	33008	11371	34.5	9628	29.2
Other GI (150-152, 155-159)	27483	4722	17.2	3032	11.0
Respiratory organs (162-165)	16520	6523	39.5	5667	34.3
Lung cancer(162)	13152	5820	44.3	5192	39.5
Other (163-165,190,192-194)	2294	578	25.2	428	18.7
Bone, connective tissue, skin and breast (170-175)	35697	3134	8.8	1674	4.7
Melanoma/sarcoma (170-172)	3314	670	20.2	515	15.5
Breast (174)	30118	2315	7.7	1052	3.5
Genitourinary organs (179-189)	39475	5125	13.0	3050	7.7
Ovary (183)	3737	519	13.9	335	9.0
Prostate (185)	9782	1328	13.6	835	8.5
Metastases (196-199)	10086	4837	48.0	4215	41.8
Lymphoma/leukemia (200-208)	9403	2114	22.5	1334	14.2

**Figure 1:**
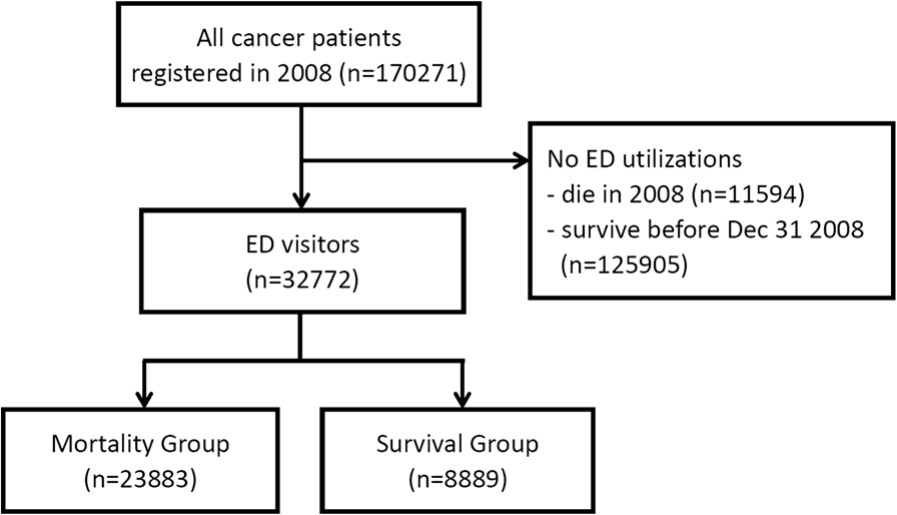
Flow diagram of this study.

We used the cancer data file and death registrations from all insured beneficiaries to select the study subjects from Jan 1 2008 to Dec 31 2008. We investigated the ED utilizations of all cancer survivors and decedents. A total of 177,721 patients with a cancer diagnosis were identified, 35,477 cancer patients who died in 2008 and 142,244 survivors who were alive in 2008. 125,905 cancer survivors and 11,594 cancer decedents who died in 2008 without making an ED visit in the one-year observation period were excluded. For comparison without a lead time bias, 7,450 subjects in the survival group were also excluded as their survival status was missing or they did not survive or were not identified in the cancer dataset at Dec 31 2008. Thus, 170,271 patients with a cancer diagnosis were eligible and the ED visitors numbered 32,772. Among the ED visitors, the surviving cases totalled only 8,889. The other 23,883 cancer patients who died in 2008 were retrospective to investigate the whole one year records before they died. The flowchart of this study is shown in Figure 1.

### Statistical analysis

Descriptive statistics are represented as numbers of cases, percentages, and means with standard deviation (SD). Categorical data were analyzed using the Chi-square test. The Mantel-Haenszel Chi-square test was used to test for trends. The independent t-test and 95% confidence intervals (95% CIs) were used to analyze the significant differences in ED utilization between the two subgroups. All analyses of data were conducted using Microsoft SQL Server 2012 (Microsoft Corporation) and statistical calculations were performed using the Statistical Package for Social Sciences for Windows (SPSS 22.0).

## Results

### Characteristics of the cancer population

The characteristics of the cancer patients and emergency cases that had used emergency department (ED) services before death are described in Table 1. A total of 170,271 patients with malignant cancer were identified, of whom 84,215 were male and 86,056 were female. Of those, the eligibility criteria identified 32,772 (19.2%) patients who had utilized an ED and 23,883 (14.0%) who died during the study period. The most frequent types of cancer in 2008 were digestive and peritoneum cancers (34.8%), followed by breast cancer (17.7%) and cancers of the head and neck (13.3%). However, the most common medical problems leading to an ED visit were found to be metastases (ICD-9 codes 196-199), lung cancer (code 162), and digestive and peritoneum cancers (codes 150-159). The top three mortality rates of cancer patients who visited an ED within 6 months of the end of life were those with metastases (41.8%), lung cancer (39.5%) and colorectal cancer (29.2%). Table 1 also shows the age and gender distributions of the enrolled subjects. Approximately 20% of the cancer patients made an ED visit in a year, the majority being male patients (23.8%), older patients, and those near the end of life. The mean age of the mortality group was 67.0 (±14.4) years, and was significantly greater than that of the survival group (*p* < 0.001).

The characteristics of the cancer patients in 2008 are listed in Table 2, stratified by survival status. 35,477 (20.8%) cancer patients who died during the study period were classified into the mortality group; the other 134,794 (79.2%) survivors were alive in 2008 and were classified into the survival group. Among the mortality group, 23,883 dying patients used the majority of the ED resources, with 58,791 ED visits (81.5%), and the mean number of visits was 2.46 per person (95% confidence interval, CI: 2.43 to 2.49) in the last six months of life. The prevalence of ED utilization was 67.3% (95% CI: 66.8% to 67.8%) before death, which was significantly higher than that of the survival group (with a prevalence of 6.6%, 95% CI: 6.5% to 6.7%). Among the survival group, the average number of ED visits was 1.51 per person (95% CI: 1.47 to 1.55) within the 6-month observational period, which was significantly lower than that of the mortality group (*p* < 0.001).

**Table 2:** **Utilization of emergency medical services among the cancer population in Taiwan**

	**Mortality group (n = 35477)**	**Survival group (n = 134794)**	***p*** **-value**
ED-visiting subjects	23883	8889	
Age, mean (S.D.)	67.0 (14.4)	59.5 (14.7)	<0.001
Gender, n (%)			
Female	8332 (34.9)	4403 (49.5)	<0.001
Male	15551 (65.1)	4486 (50.5)	
Types of hospital, n (%)			
Community hospital	8980 (37.6)	3138 (35.5)	<0.001
Medical centers or local hos.	14903 (62.4)	5701 (64.5)	
ED hospitals with hospice, n (%) ward			
No	9332 (39.1)	3366 (38.1)	0.052
Yes	14551 (60.9)	5473 (61.9)	
Location of hospital, n (%)			
Rural	3624 (15.2)	1071 (12.1)	<0.001
Urban	20259 (84.8)	7768 (87.9)	
Prevalence of ED visit (95% CI)	67.25 (66.8-67.8)	6.60 (6.5-6.7)	<0.001
Admission after the ED visits^a^, n (%)	1160 (4.9)	210 (2.4)	<0.001
Distributions of ED visits triages^b^			
Level I, count(%)	5647 (11.3)	575 (4.3)	<0.001
Level II, count(%)	23521 (47.0)	5508 (41.3)	
Level III, count(%)	19444 (38.9)	6892 (51.7)	
Level IV, count(%)	1387 (2.8)	366 (2.7)	

^a^Mean of those admission days were shown in text; Medical Cost, averaged number of ED visiting times, and prevalence of ED visit were shown in the text.

^b^Excluding missing data, the distributions of traditional ED triaged were presented because a new 5-level triage scale had been proceed in Taiwan after 2010.

The patients in the mortality group who had used ED services had a more severe triage (p < 0.001), and higher admission rate (4.9% vs 2.4% compared to the other group, p < 0.001) that could lead to subsequent aggressive interventions and more treatment-related expenditure and greater length of stay in their last 6 months of life. Their total medical expenditure was significantly higher than the expenditure for the surviving cancer patients during the same observational period (US$7,442.4 vs. US$4,243.6, *p* < 0.001). In average, the length of stay 3.61 days (±3.47) was estimated for the mortality group, which was significantly more than the length of stay for the survival group (mean length of stay: 2.91 ± 1.47). Each kind of medical expenditure in emergency care, including diagnosis, treatment, drug costs and partial payment by themselves, each facet was observed in the mortality group approximately 1.4 to 2-fold higher than in the survival group (p < 0.001).

Dying patients could make ED visits for the relief of symptoms in the nearest community hospital rather than medical centers under emergency conditions. The odds ratio of the use of community hospitals for ED visits was significant at 1.10 (95% CI: 1.04-1.15) in the mortality group patients. The rural location of the hospital (OR = 1.30, 95% CI: 1.21-1.40) was also correlated with the ED utilization in emergency conditions, but hospitals with a hospice ward were not significantly related (OR = 1.04, 95% CI: 0.99-1.10) among the cancer population.

### Comparisons of diagnostic categories of ED visits between cancer survivors and cancer decedents

ED visits for cancer-related problems were identified from the cancer dataset of the National Health Insurance Research Database (NHIRD). Of 211,054 ED diagnostic problems during a 6-month period, there were 147,192 diagnoses in the mortality group and 63,862 diagnoses in the surviving group. The ICD-9-CM diagnoses codes were categorized as described in Table 3. Besides the primary cancer diagnosis, the 15 most common diagnoses for ED visits were ranked by frequency and analyzed by the Chi-square test. The highly-ranked diagnoses differed slightly between the two groups. The first-ranked problem for visiting an ED in both groups was related to symptoms, signs, and ill-defined conditions (approximately 35%). Visits related to the digestive system ranked second in both groups (18.5% - 19.0%). However, the third-ranked problem among the cancer survivors and decedents differed. The respiratory system ranked third (12.3%) among the mortality group during the last six months of life, while genitourinary problems (11.2%) ranked third in the cancer survivors. Besides symptoms, signs, and the primary cancer, the following chief problems in the mortality group were observed significantly less often than in the surviving group: genitourinary system, circulatory system, nervous system, mental disorders, musculoskeletal system and congenital abnormalities.

**Table 3:** **Distributions of ED utilization stratified by diagnostic category and survival status (n = 32,772)**

**Summed disease categories of all ED utilizations**		**Mortality group (** ***n*** **= 23,883)**	**Survival group (** ***n*** **= 8889)**	***p*** **value**
**ICD_9 codes diagnostic category**		**(%)**	**(%)**	
*001-139*	Infectious and parasitic diseases	4.35	3.35	<0.001
*140-239*	Neoplasms	100	100	1.000
*240-279*	Endocrine, nutritional and metabolic diseases and immunity disorders	8.20	6.45	<0.001
*280-289*	Diseases of the blood and blood-forming organs	4.44	3.07	<0.001
*290-319*	Mental disorders	0.76	1.63	<0.001
*320-389*	Diseases of the nervous system and sense organs	1.48	2.38	<0.001
*390-459*	Diseases of the circulatory system	6.55	7.86	<0.001
*460-519*	Diseases of the respiratory system	12.31	8.40	< 0.001
*520-579*	Diseases of the digestive system	19.01	18.54	0.147
*580-629*	Diseases of the genitourinary system	7.44	11.17	< 0.001
*680-709*	Diseases of the skin and subcutaneous tissue	1.32	2.66	< 0.001
*710-739*	Diseases of the musculoskeletal system and connective tissue	2.21	2.64	< 0.001
*740-759*	Congenital abnormalities	0.08	0.13	0.018
*760-779*	Certain conditions originating in the perinatal period	0.02	0.02	0.606
*780-799*	Symptoms, signs, and ill-defined conditions	34.52	39.25	< 0.001
*800-999*	Injury and poisoning	2.34	4.17	< 0.001

Of all ED utilizations among all cancer patients, the second top ranked problem was diseases of the digestive system, but there was no statistically significant difference in ED utilization between the mortality group and the survival group (19.0% v.s. 18.5%, *p* = 0.147). Among the mortality group, patients with digestive problems had a high number of ED visits during their last six months of life in Taiwan.

**Figure 2:**
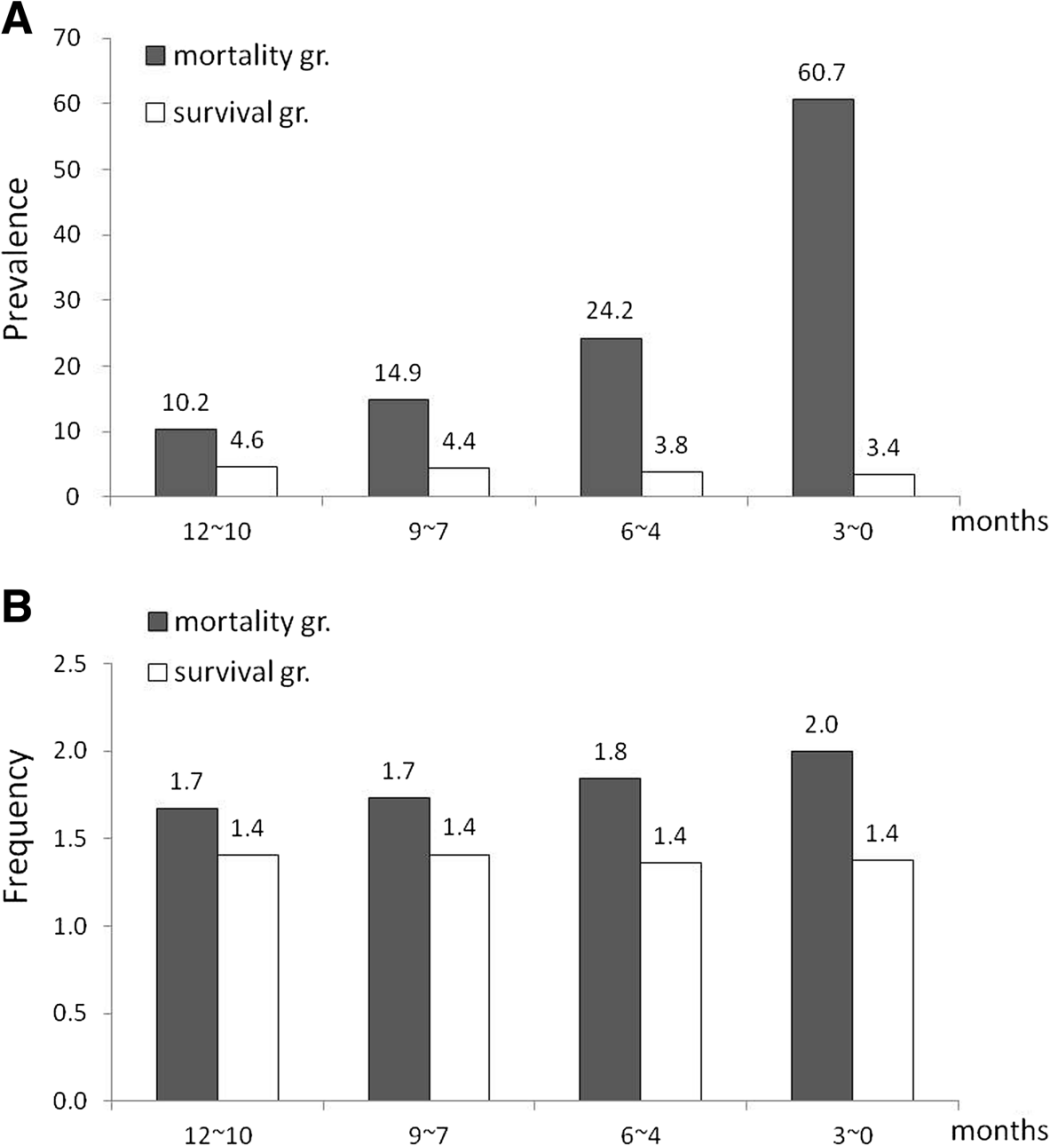
ED utilizations in the mortality and survival groups. **A**. Prevalence. **B**. Frequency of ED visiting times.

Analyzing our cancer data, the prevalence of ED utilization for cancer patients in Taiwan is illustrated in Figure 2. The distributions of ED utilization in various months in the two compared groups (mortality vs. survival groups) were obviously different in every three-month period in 2008. Among the mortality group, a higher prevalence of ED visits was found in the last 12-month period. Furthermore, when the time was closer to the end of life, ED visits became more frequent in the cancer decedents. A Mantel-Haenszel extension of the chi-square test for trend was significant (*p* < 0.001).

## Discussion

Palliative care is an essential and fundamental component of cancer care [22]. In end-of-life care, emergency staffs (including physicians and nurses) are asked to receive hospice patients. ED staff need to recognize the complexities involved for the best management of a growing population of hospice patients. This study addressed the suffering of EOL patients using a nationwide dataset, and also described the visits of surviving patients with terminal cancer who presented to EDs [23]. Although palliative care is generally offered with the standard services in various settings, systematic characteristics influence the ability to implement the delivery of care for terminally ill patients [24]. Based on this evidence, an integrated community care service model should be developed, which integrates palliative care throughout the cancer trajectory [22].

Health system characteristics and patient cultural factors may play roles in the observed differences [6]. Several studies in Western countries have emphasized that hospice care at home is best for dying patients with cancer rather than aggressive treatment. However, Taiwan health insurance covers almost all medical services provided in hospitals for cancer patients, and therefore there are fewer economic barriers in end-of-life care. The barriers to ED utilization are decreased for cancer patients owing to healthcare fee remission. A study evaluating the medical utilization of the Taiwan NHI system revealed that on average, a person had 13.4 physician consultations and visited 3.9 healthcare facilities in a year, and 17.3% of the studied cohort had visited different healthcare facilities on the same day [25]. A co-payment method was introduced into Taiwan’s healthcare system, which could add some economic load and decrease inappropriate use and overuse of medical resources. A study analyzing the correlation between NHI-defined catastrophic illness and remitting medical expenditure revealed that patients with any certificated catastrophic illness tended to have significantly more ED visits and higher ED costs due to untypical medical complaints [16]. Furthermore, cancer care could potentially tend to become aggressive, as shown by the frequent ED visits and admissions to intensive care units at patients’ end of life [5,13]. Advanced care planning emphasizes value-based care and enables further development towards hospice and palliative care [24].

The primary purpose of this study was to explore the prevalence of ED utilization and the expenditure on emergency care among the Taiwanese cancer population. A greater emergency medical expenditure at the end of a six-month period was observed in the mortality group, which included treatment-associated expenditure and drug-associated expenditure in Taiwan’s EDs. The average costs were significantly higher in the mortality group than in the survival group (US$7,442.4 vs. 4,243.6; *p* < 0.001). The high utilization and medical costs were similar to those reported in the US and Canada [26]. One study focused on patients who died of cancer in 2001; 27.6% had at least one ER visit and 5.4% had an ICU visit in the last two weeks of life, and Barbera [15] indicated that the high proportion of intensive therapies for cancer patients very near to death represent poor-quality end-of-life care. Another similar result was also found in North Carolina in 2008: there were 194,017 ED visits by 76,759 patients with cancer, and among these visits, those paying the most were with Medicare (for 52.4%) and Medicaid (for 12.1%). Hence, cancer treatment costs are increasing at an unprecedented rate [16,26]. The economics of health care need to be emphasized, and home-based palliative care needs to be considered in order to decrease the emergency expenditure and number of visits at the end of life [27,28]. Palliative care is one component of rural practice that requires interprofessional collaboration. Members of the care team need to have a sense of accountability and mutual respect. Thereby the autonomic team of rural practitioner devote their time to palliative care. That would be one of potential reasons that access to care and service utilization might be employed [29].

Age and gender effects were observed in end-of-life ED utilization. In the present nationwide population study, and some studies in the US and Canada, patients were found to be more likely to receive aggressive cancer care at the end of life if they were male, younger, lived in rural regions, had a higher level of comorbidity, or had breast, lung, or hematologic malignancies [6,30]. However, another study debated whether cancer-related ED visits are more likely to be made by male patients and those of an older age [31].

The results of Leak’s study also indicated that the chief complaints among cancer ED visitors were in those dying of respiratory and gastrointestinal symptoms and those with a mental status change according to the 37,760 cancer-related ED visits contained in the North Carolina Disease Event Tracking and Epidemiologic Collection Tool [31]. The results were similar to the reason distributions of the identified categories of patients with cancer presented in the current study. Cardiopulmonary arrest, respiratory distress, and shortness of breath are well-known symptoms among end-of-life cancer patients [32]; however, we need to be aware that abdominal pain and gastrointestinal issues are also some of the most frequent problems for cancer ED visits during the final six months of life [16]. The results of Burge’s study in Canada indicated that patients with a low continuity of care made 3.9 more ED visits than those experiencing a high continuity of care. The significant association between continuity of care and ED visits at the end of life among cancer patients needs to be emphasized [28]. ED visits can be prevented with appropriate home support and education and a focus on symptom management [9,33], and keeping patients more comfortable at home under palliative care indicates good-quality end-of-life cancer care [12,34].

The strength of the present study was the nationwide epidemiological survey to analyze the end-of-life care for the Taiwanese cancer population. Some limitations exist: if a patient suffered prolonged hospitalization in his or her last 6 months of life, we may underestimate the ED utilization. An additional limitation of claims data mining is that there is a loss of detail. Possible confounders were not controlled in the present study, including socio-economic influences and family support, which were unable to be extracted from the epidemiological data. If patients do not have valid insurance, they may die in distress. Further investigation using an integrated community care model throughout the cancer trajectory survey is recommended.

## Conclusion

Palliative care is an important issue for Taiwan cancer patients and deserves more attention. Population-based data can further provide evidence to support the development of a cancer policy and EOL care services. Our study provided population-based evidence of ED visits by patients with cancer in Taiwan. The results also provided empirical evidence regarding what EOL cancer patients perceive as being their needs. Patients with malignant cancer diagnoses had significantly more ED visits and higher ED medical costs. An understanding of the problems leading to such visits could be useful in preventing the overuse of ED visits to improve the quality of EOL care.
